# Different Electrophysiological Responses to Informative Value of Feedback Between Children and Adults

**DOI:** 10.3389/fpsyg.2018.00346

**Published:** 2018-04-03

**Authors:** Bin Du, Bihua Cao, Weiqi He, Fuhong Li

**Affiliations:** ^1^Research Center of Brain and Cognitive Neuroscience, Liaoning Normal University, Dalian, China; ^2^School of Psychology, Jiangxi Normal University, Nanchang, China

**Keywords:** feedback, informative value, development, ERP, rule induction

## Abstract

The ability to learn from feedback is important for children’s adaptive behavior and school learning. Feedback has two main components, informative value and valence. How to disentangle these two components and what is the developmental neural correlates of using the informative value of feedback is still an open question. In this study, 23 children (7–10 years old) and 19 adults (19–22 years old) were asked to perform a rule induction task, in which they were required to find a rule, based on the informative value of feedback. Behavioral results indicated that the likelihood of correct searching behavior under negative feedback was low for children. Event-related potentials showed that (1) the effect of valence was processed in a wide time window, particularly in the N2 component; (2) the encoding process of the informative value of negative feedback began later for children than for adults; (3) a clear P300 was observed for adults; for children, however, P300 was absent in the frontal region; and (4) children processed the informative value of feedback chiefly in the left sites during the P300 time window, whereas adults did not show this laterality. These results suggested that children were less sensitive to the informative value of negative feedback possibly because of the immature brain.

## Introduction

In the development of infancy and childhood, the ability to learn from inappropriate behavior and flexibly switch to the proper performance is important for their daily school life (Dajani and Uddin, 2015; Hauser et al., 2015; Wolff et al., 2016; Shnitko et al., 2017). The most typical paradigm to study cognitive flexibility is rule-learning task. In these types of tasks, the informative value of feedback plays an important role in searching the correct rules, which provides information that guiding the next selection (Barceló, 2003; Zanolie et al., 2008b; Mies et al., 2011; Wang et al., 2015). For instance, in the Wisconsin Card Sorting Test (WCST), participants need to find the correct cards by changing different rules. When participants received negative feedback, they needed to shift to a new rule (Milner, 1963; Heaton et al., 1993; Miller, 2000; Dias et al., 2015; Farreny et al., 2016; Ohyama et al., 2016).

Several studies using rule learning or performance monitoring tasks have revealed a frontal positivity at approximately 180 and 300 ms, which was called the P2 component (Potts, 2004; Groen et al., 2007; Zottoli and Grose-Fifer, 2012). P2 has been interpreted as an index of attention assignation (Barceló, 1999; Potts et al., 2006; San Martín et al., 2010). In a gambling task, San Martín et al. (2010) investigated the differences in feedback for distinct types of valence, size, and probability. The results showed that winning was associated with a greater P2 amplitude than losing. Moreover, P2 was also modulated by different probability or size of reward. It was suggested that participants paid more attention to the feedback that followed high probability or greater size of the offered rewards (Yu et al., 2011; Kreussel et al., 2012). However, other studies showed that the P2 component reflected the process of feature detection (Bigman and Pratt, 2004; Chen et al., 2007; Pincham, 2014). For example, Bigman and Pratt (2004) studied the time course and nature of category induction in 11–13-year-old adolescents. The results showed that P2 latency was longer in response to the first stimulus than to the second stimulus. They interpreted that the processing of following stimuli was benefit from the context of the first stimulus, so it was more efficient, and the features for the following stimulus could be analyzed more quickly.

Previous studies on feedback have demonstrated that feedback-related negativity (FRN), also called N2, likely generated in the anterior cingulate cortex, was induced when participants received feedback (Wild-Wall et al., 2009; van der Helden et al., 2010; Martínez-Velázquez et al., 2015; Ferdinand et al., 2016; Gu et al., 2017). In comparison with positive feedback, negative feedback elicited a more negative FRN (Nieuwenhuis et al., 2005; van der Veen et al., 2008; Hämmerer et al., 2011; Opitz et al., 2011; Walsh and Anderson, 2012; Arbel et al., 2013). There is a large corpus of evidence suggesting that FRN reflects the processing of performance monitoring (Yeung et al., 2004; Eppinger et al., 2009; San Martín, 2012). Eppinger et al. (2009) have used a probabilistic learning task to examine the processes of performance monitoring for children aged 10–12 years and adults aged 19–24 years. It was demonstrated that, despite the feedback validities, both groups exhibited a larger FRN to the negative feedback than to the positive feedback. Moreover, the FRN amplitude in children was larger than that in adults. The results showed that participants utilized the external error feedback to adjust their performance, especially for children. However, when task emphasized other related aspects, such as expectancy, and valence was weakened, FRN was no longer sensitive to feedback valence but was sensitive to expectancy (Ferdinand et al., 2012; Ferdinand and Kray, 2014).

P300 was also modulated by feedback valence and generally larger for positive feedback (Hajcak et al., 2005). In contrast, some studies demonstrated that negative feedback induced a larger P300 than positive feedback (Yeung et al., 2005; Groen et al., 2007).

To our knowledge, only a handful studies have directly explored the informative value of feedback. Barceló (2003) used a variant task-switching paradigm of WCST, in which feedback cued unpredictable shifts (i.e., from “sort cards by color” to “sort cards by shape”). They found that feedback signaling the set-shifting induced a P3b, which was sensitive to the number of rules held in memory, and it was suggested that P3b may reflected the processing of the informative value of feedback. Lange et al. (2015) investigated the electrophysiological differences between informative feedback and redundant feedback. They found that the former elicited an obvious P300 component in the frontal areas and interpreted that the informative feedback provide important information for successful task performance. Walentowska et al. (2016) compared the feedback that were relevant (i.e., informative) to the task performance to the irrelevant one. The results showed that feedback in the relevant blocks induced a larger P300 than did the irrelevant blocks.

Although the abovementioned studies have investigated the neural mechanism underlying the processing of the informative value of feedback, its developmental characteristics are still not well understood. Are children more likely to acquire information from negative feedback or positive feedback? There is no unified conclusions to this question. By using a probabilistic learning task, Eppinger et al. (2009) found a larger FRN and a reduced ERP learning effect on positive feedback for children. They interpreted that children were more sensitive to negative feedback and less able to learn from positive feedback. Similarly, Hämmerer et al. (2011) found that children were more sensitive to loss (negative feedback) than to gain (positive feedback). However, others studies found that children additionally show higher sensitivity to positive feedback (Lange-Küttner et al., 2012; Hentschel et al., 2016; Zhuang et al., 2017). For instance, Lange-Küttner et al. (2012) investigated the influence of feedback by a sequence learning task which involves deductive reasoning on a larger sample of 8- to 11-year-old children. The task included stochastic feedback and deterministic feedback. The results revealed that children learned more when they received positive feedback than negative feedback, regardless of the certainty of feedback. Moreover, the learning effects of negative feedback that increased with age were only observed for the latter task and started at age 9 years. Hentschel et al. (2016) used the same task to study the boys with Autistic Spectrum Disorder (ASD) and healthy boys. They found that both groups learned more from positive feedback than from negative feedback. Other studies that using behavioral methods, fMRI, or heart rate recordings coherently found that, when presented with negative feedback, children adjusted their behavior less successfully than adults did (Crone et al., 2004a; Huizinga et al., 2006; van Duijvenvoorde et al., 2008; Van Duijvenvoorde et al., 2013). However, these studies did not disentangle the informative value from the valence of feedback.

It is necessary to note that, in some feedback learning tasks, feedback seemingly contained some informative value, but the information cued by the feedback is ambiguous. For example, in the probability learning task (Cohen and Ranganath, 2007; Bellebaum and Daum, 2008; Hauser et al., 2015), participants could relied on different feedbacks to keep or change their behavioral strategy, but the informative value was not very clear when feedback was presented. That is, negative feedback that appeared once in a trial did not necessarily indicate that the rule had changed; participants could be sure that the rule had changed only when negative feedback was displayed in more trials.

Most recently, Ferdinand et al. (2016) used a time estimation paradigm to try to disentangle the contributions of valence and expectancy in feedback processing. In the task, participants can adjust their time estimation according to the informative value of feedback. When they received an unexpected negative feedback in a trial because of the too fast or too slow response, they would adjust their reaction in the next trial to avoid get a negative feedback again. The ERP results indicated that during P300 time window, both children and adolescents could took advantage of feedback expectancy which was equaled to the informative value to adjust their performance. Interestingly, adolescents with better behavioral adaptation had a more frontal P300 expectancy effect, while children did not show this effect, implying that frontal P300 might be associated with the processing of the informative value of feedback.

The purpose of this study is to investigate whether children learn more from positive or negative feedback, and to explore the age differences of brain activation underlying the processes of the informative value of feedback. It has been argued that children of approximately 9 years old can successfully abstract types of sameness (Smith, 1989), so we adopt a new paradigm called as rule induction task (RIT), which is similar to WCST. RIT allows us to disentangle informative value from feedback very well. In the RIT, one target stimulus and four testing stimuli were displayed on the screen. Participants were asked to match the testing stimuli to the target according to a hidden rule. After their first match, they would receive a positive or negative feedback. In order to distinguish the informative value from the valence of feedback, we designed a control condition in which only the valence of feedback was involved. Subtracting the ERPs evoked by the feedback of RIT from the ERPs evoked by the feedback of control can unfold the pure neural responses to the informative value of feedback.

Based on previous studies (Diamond, 2002; Sowell et al., 2004; Crone et al., 2006a,b; Durston et al., 2006; Luciana and Collins, 2012; Ferdinand and Kray, 2014; Gennatas et al., 2017), which suggested that the biological structure and the psychological function of the frontal lobes differed markedly between the children and adults, we predicted that compared to adults, children might show a different pattern of ERP response to the informative value of feedback. Particularly, children might be less sensitive to the informative value of negative feedback, since they were more likely to be driven by positive feedback (Somsen, 2007; van der Veen et al., 2008; Zhuang et al., 2017). In addition, based on the results of the two studies in our laboratory (Li et al., 2011; Huang et al., 2013), we predicted that the processes of informative value of feedback for children might be observed at the left sites.

## Materials and Methods

### Participants

A total of 42 subjects from two age groups (23 children and 19 adults) participated in this study. Seven subjects were excluded from ERP analysis because of excessive eye movement or artifacts. The remaining 16 children (9 females and 7 males) were aged between 7 and 10 years old (mean age: 8.69 years; *SD*: 0.77 years). The adult group consisted of 19 subjects (14 female and 5 male) between 19 and 22 years old (mean age: 21.74 years; *SD*: 2.40 years). All participants were right-handed and had normal or corrected-to-normal vision. They reported no history of neurological or psychiatric diseases. All children’s parents and the adult participants provided both verbal and written consent for participation of this study, and the conduction of the study was approved by the Ethics Committee Jiangxi Normal University (China).

### Design and Materials

A feedback-based RIT was used. In each trial, subjects were presented with five geometrical stimuli displayed at the center of a 19-inch screen (CRT monitor), wherein the target was on the left side and the testing stimuli were on the right side. Within each trial, stimuli were varied in shape (square, triangle, echelon, round, rectangle, hexagon), color (red, yellow, green, blue, beige, brown), and pattern (grid, horizontal stripe, wave, diagonal, vertical stripe, solid diamond). The distance between the participants’ eyes and the screen was approximately 1.2 m. The horizontal and vertical visual angles were <5° for the stimuli presented on the screen.

**FIGURE 1:**
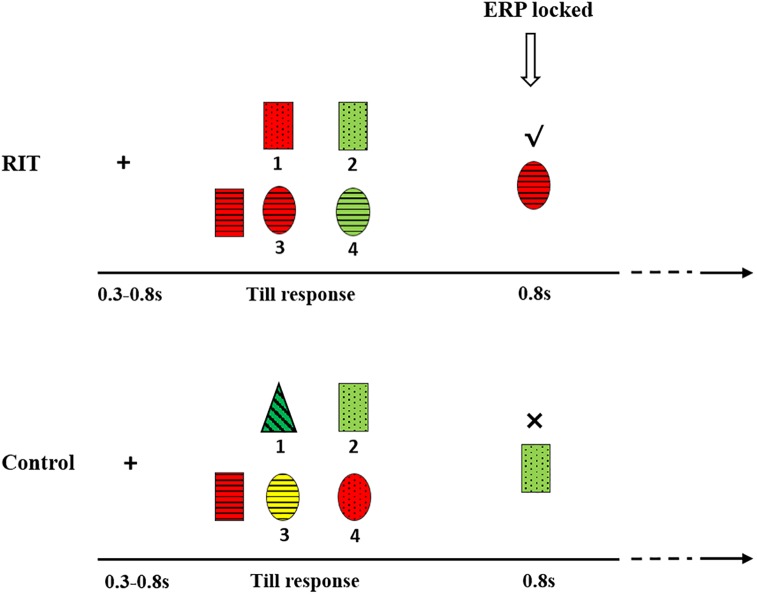
Experimental procedure and sample stimuli. The above is an example of the RIT and the below is an example of the control condition.

Two conditions, the RIT and the control were designed. In both conditions, children were asked to find candies from the testing stimuli. In the RIT, the left target stimulus was a candy and two of the four testing stimuli were candies, while the other two were not (**Figure 1**). In each trial, all candies (i.e., one target and two testing stimuli) shared one common attribute. For example, all candies have horizontal stripes (**Figure 1**, bottom panel). There were three perceptual dimensions, and subjects did not know which perceptual dimension was associated with the identification of a candy. After trial and error, they could find the rule regarding the category to which the candies belonged. The participant’s task was to find the two candies among the testing stimuli by pressing the corresponding number below them. Participants were encouraged to find the candies with the fewest possible errors. In the RIT, participants’ first choice was based on a guess. However, children could use the informative value of the first feedback to decide which testing stimuli should be selected the second time. On each choice, a check mark would appear on the top of the selected one if it is a candy; if not, a fork mark would appear.

In the control condition, the left stimulus was also a candy, but there was only one candy among the testing stimuli. Participants either luckily found the candy (positive feedback) or failed (negative feedback). Therefore, in the control condition, there was no informative value of the feedback and instead only valence was present.

The two conditions were presented in two sessions separately. For half subjects, they completed the RIT first, and then the control. For other half subjects, the order was reversal.

### Procedure

First, a fixation “+” was presented in the center of the screen for 300–800 ms, followed by a blank screen for 200–300 ms. Five stimuli were simultaneously presented until key pressing for the participant’s response. A blank screen for 700–900 ms was then presented. After the blank screen, the stimulus which subjects chose was then presented for 800 ms, followed by a blank screen for 300–500 ms (**Figure 1**).

In the adult group, the RIT was organized in eight blocks and each block contained 30 trials. The control was organized in seven blocks and each block contained 30 trials as well. The total time for adults to finish the two sessions was about 75 min. To avoid fatigue in children, total trials were reduced about 13%.

### Electrophysiological Recording and Analysis

Electrophysiological activity was recorded with a 64-channel electroencephalogram (EEG) recording system (A.N.T. eego^TM^ software, Germany) with CPz as a reference electrode. A ground electrode (GND) was placed on the medial aspect of the frontal region between Fz and Fpz, as well as AF3 and AF4. The vertical electro-oculogram (VEOG) was recorded with electrodes placed above and below the left eye, and the horizontal electro-oculogram (HEOG) with electrodes placed by the right side of the right eye and the left side of the left eye. The EEG and EOG were amplified using a 0.05–100 Hz bandpass filter and continuously sampled at 500 Hz/channel. All inter-electrode impedance was maintained below 5 kΩ.

Raw EEG data were processed offline using BrainVision Analyzer version 2.0 (Brain Product GmbH, Gilching, Germany). For data analysis, ERPs time-locked to the onset of the first feedback were re-referenced algebraically to the average of the left and right mastoids. The EEGs were digitally filtered with a 0.01–30-Hz bandpass with a 50-Hz notch filter, before ocular correction (Jung et al., 2000). ERPs for all correct trials were then segmented into 1000-ms epochs surrounding stimulus (first feedback) presentation and baseline-corrected with 200 ms at pre-stimulus. Trails contaminated with EOG artifacts (mean EOG voltage exceeding 100 μV) or those with artifacts due to amplifier clipping, bursts of electromyographic (EMG) activity, or peak-to-peak deflection exceeding 100 μV were excluded from the average. The artifact-free EEG was averaged separately for each condition.

Within each trial of the RIT, there were two types of stimuli: two-attributes-shared stimuli and one-attribute-shared stimuli. The former shared two attributes (e.g., shape and color) with the target, while the latter shared only one attribute with the target. Behavioral results showed that both children and adults preferably choose the two-attributes-shared stimuli (the mean proportion was 79% among children and 87% among adults) over the one-attribute-shared stimuli. The numbers of trials in which one-attribute-shared stimuli chosen were rather few for both the groups. Thus, only ERPs evoked by the feedback following the two-attributes-shared stimuli trial were analyzed.

Amplitudes were measured with respect to mean voltages during the 200-ms pre-stimulus interval. In the adult group, five ERP components were identified over most of the scalp, including N1 (100–150 ms), P2 (170–260 ms), N2 (270–340 ms), P3 (340–440 ms), and LPC (550–800 ms). In the child group, four ERP components were found: N1 (120–170 ms), P2 (180–270 ms), N2 (290–490 ms), P3 (300–400 ms), and LPC (500–700 ms). The mean amplitudes were measured for all ERP components and condition-specific effects were found in most of the electrode sites. Thus, the following 18 electrodes were chosen for statistical analysis (left-frontal: F3, F5; middle-frontal: F1, F2; right-frontal: F4, F6; left-central: C3, C5; middle-central: C1, C2; right-central: C4, C6; left-parietal: P3, P5; middle-parietal: P1, P2; right-parietal: P4, P6).

Amplitudes were analyzed using a 2 (age: children, adults) × 2 (valence: positive, negative) × 2 (condition: control, RIT) × 3 (caudality: frontal, central, parietal) × 3 (laterality: left, middle, right) analysis of variance (ANOVA). Because there was no P3 component over the frontal scalp in the child group, so we separately analyzed the P3 differences between adult and child groups. And in the child group, only the parietal electrodes (P1, P2, P3, P4, P5, P6) during the P3 (300–400 ms) time window were analyzed. For all analyses, the *p*-value was corrected for deflections according to the Greenhouse–Geisser method.

## Results

### Behavioral Results

**FIGURE 2:**
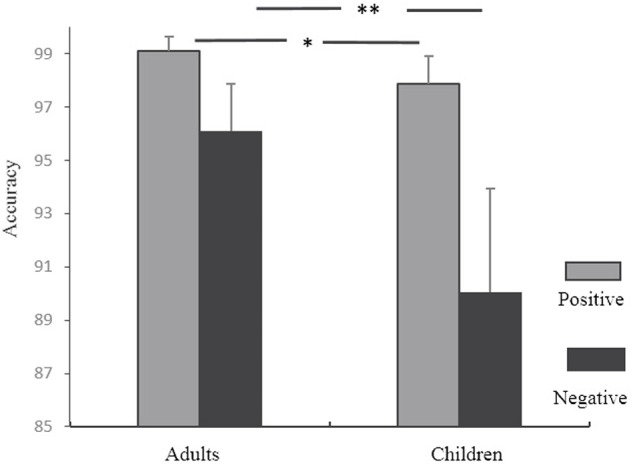
Searching accuracy under different feedbacks following the first selection in RIT. ^∗∗^*p* < 0.01; ^∗^*p* < 0.05.

The accuracy in rule searching for each subject in the RIT was analyzed. We only analyzed behavioral performance when children first chose the two-attributes-shared stimuli. There were several correct logical routes and wrong routes in searching, and the searching accuracy here was defined as the correct logical routes (see the Supplementary Materials for details). For example, when participants chose the two-attributes-shared stimuli for the first time and received a positive feedback, a trial was marked wrong if the second selection had no common features with the first selection. When they received the first negative feedback after choosing the two-attributes-shared stimuli, then on the second selection, if the selected stimuli shared no common features with the first selection, this trial was marked correct. The searching accuracies were submitted to a 2 feedback (positive, negative) × 2 age (children, adults) ANOVA with repeated measurements. Age was treated as a between-subject variable while feedback was treated as a within-subject variable. A significant main effect of feedback was found, *F*(1,33) = 34.64, *p* < 0.001, η^2^ = 0.51. Accuracy for positive feedback was higher than that for negative feedback. A significant main effect of age was also found, *F*(1,33) = 10.68, *p* < 0.01, η^2^ = 0.25. Accuracy was higher for adults than for children. The interaction of age and feedback was significant, *F*(1,33) = 6.44, *p* < 0.05. As shown in **Figure 2**, for children, the mean accuracy under positive feedback (*M* = 97.87%, *SD* = 0.02) was significantly higher than that for negative feedback (*M* = 90.06%, *SD* = 0.08), *F*(1,33) = 6.13, *p* < 0.05. In the adult group, the mean accuracy for positive feedback (*M* = 99.21%, *SD* = 0.01) was higher than that for negative feedback (*M* = 96.11%, *SD* = 0.03), *F*(1,33) = 32.68, *p* < 0.001. In both types of feedback, the between-group difference was significant (*p* < 0.05).

### Electrophysiological Results

#### P2 Component

The age and valence effects were both non-significant (all *p*s > 0.05). A significant main effect of condition was found [*F*(1,68) = 22.13, *p* < 0.001, η^2^ = 0.25]. The P2 amplitude for the RIT was more negative than for the control condition (**Table 1**).

**Table 1:** Results of ANOVAs on the mean amplitude for P2, N2, and LPC components.

ERP components	Valence *F*(*p*)	Condition *F*(*p*)	Valance^∗^condition^∗^age *F*(*p*)	Valence^∗^condition^∗^laterality^∗^age *F*(*p*)
P2	3.42 (0.069)	22.13 (0.000)^∗∗∗^	0.22 (0.644)	2.67 (0.076)
N2	54.28 (0.000)^∗∗∗^	89.34 (0.000)^∗∗∗^	26.65 (0.000)^∗∗∗^	3.96 (0.024)^∗^
LPC	1.62 (0.208)	55.15 (0.000)^∗∗∗^	3.71 (0.058)	2.21 (0.118)

^∗∗∗^*p < 0.001; ^∗∗^*p* < 0.01; ^∗^*p* < 0.05*.

**FIGURE 3:**
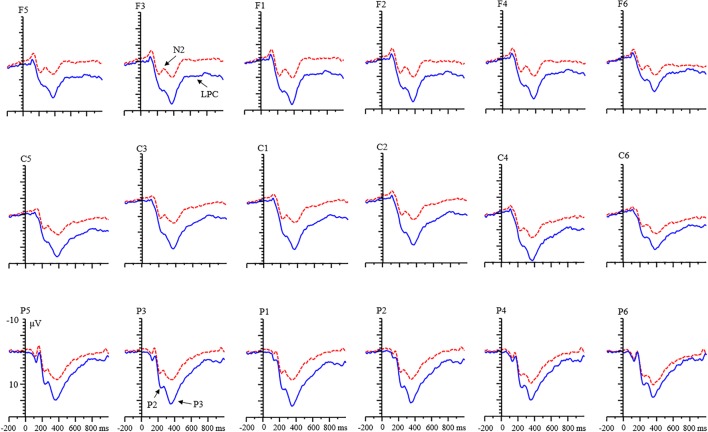
Grand averages of event-related potentials (ERPs) evoked by positive feedback for adults.

There was an interaction among valence, condition, age, and laterality [*F*(2,136) = 4.12, *p* < 0.05, η^2^ = 0.07]. Simple effects analysis showed that (1) for adult group, the effect of valence was not found in the RIT condition in all levels of laterality (all *p*s > 0.05), but the effect of valence was found in the control condition in each level of laterality, with a larger amplitude for positive feedback than for negative feedback (all *p*s < 0.05). For the child group, the amplitude of positive feedback was smaller than that of negative feedback in the left and middle regions [left: *F*(1,68) = 18.30, *p* < 0.001; middle: *F*(1,68) = 7.92, *p* < 0.01; right: *F*(1,68) = 2.10, *p* = 0.152]. (2) Under the positive feedback, the condition effect existed in the adult group (**Figure 3**). The P2 amplitude for the RIT was smaller than that for the control condition in each level of laterality (all *p*s < 0.001). In the child group, this effect only existed in the left and middle laterality [left: *F*(1,68) = 12.62, *p* < 0.01; middle: *F*(1,68) = 10.13, *p* < 0.01; right: *F*(1,68) = 3.53, *p* = 0.065]. In the negative feedback, the effect of condition only existed in the left and middle laterality for adults [left: *F*(1,68) = 15.57, *p* < 0.001; middle: *F*(1,68) = 10.43, *p* < 0.01; right: *F*(1,68) = 1.69, *p* = 0.198]. But this effect was not found in all levels of laterality for children.

#### N2 Component

There was a main effect of age on the N2 component [*F*(1,68) = 29.96, *p* < 0.001, η^2^ = 0.31]. The N2 amplitude for the children was more negative than that for the adults. A main effect of valence was found [*F*(1,68) = 54.28, *p* < 0.001, η^2^ = 0.45]. The amplitude of negative feedback was smaller than that of positive feedback. The condition effect was also significant [*F*(1,68) = 89.34, *p* < 0.001, η^2^ = 0.57]. N2 amplitude for the RIT was more negative than that for the control condition.

**FIGURE 4:**
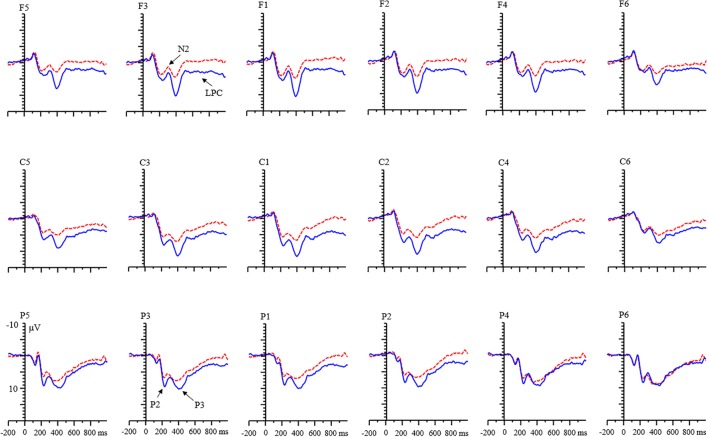
Grand averages of ERPs evoked by negative feedback for adults.

An interaction among valence, condition, and age was found [*F*(1,68) = 26.65, *p* < 0.001, η^2^ = 0.28]. Further analysis indicated that (1) in the adult group, the valence effect was found, and the amplitude for negative feedback was smaller than that for positive feedback in both condition (all *p*s < 0.01). In the child group, this effect only existed in the RIT condition [RIT: *F*(1,68) = 6.35, *p* < 0.05; control condition: *F*(1,68) = 0.07, *p* = 0.797]. (2) There was a condition effect for adult group. The amplitude of N2 for RIT was more negative than that of the control condition (all *p*s < 0.05; **Figure 4**). It was the same in the child group (all *p*s < 0.01).

#### P3 Component

In the adult group, there was a main effect of condition [*F*(1,37) = 88.09, *p* < 0.001, η^2^ = 0.70]. P3 amplitude was smaller for the RIT than for the control condition. A significant main effect of valence was found [*F*(1,37) = 50.28, *p* < 0.001, η^2^ = 0.58]. The amplitude for the positive feedback was larger than for the negative feedback (**Table 2**).

**Table 2:** Results of ANOVAs on the mean amplitude of P3 for adults and children.

ERP components	Valence *F*(*p*)	Condition *F*(*p*)	Condition^∗^ laterality *F*(*p*)
Adults	50.28 (0.000)^∗∗∗^	88.09 (0.000)^∗∗∗^	36.45 (0.000)^∗∗∗^
Children	0.004 (0.949)	7.17 (0.012)^∗^	7.79 (0.002)^∗∗^

**FIGURE 5:**
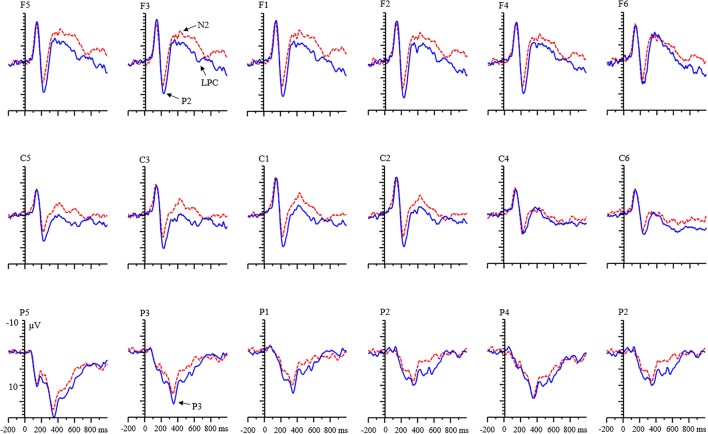
Grand averages of ERPs evoked by positive feedback for children.

In the child group, there was only a main effect of condition [*F*(1,31) = 7.17, *p* < 0.05, η^2^ = 0.19]. The P3 amplitude of RIT was smaller than that for control condition. The main effect of valence was not found [*F*(1,31) = 0.004, *p* = 0.949]. There was an interaction between condition and laterality [*F*(2,62) = 40.10, *p* < 0.001, η^2^ = 0.23]. Simple effects analysis showed that the effect of condition only existed in the left and middle laterality [left, *F*(1,31) = 12.91, *p* < 0.01; middle, *F*(1,31) = 8.29, *p* < 0.01; right, *F*(1,31) = 0.62, *p* = 0.43] (**Figure 5**). More negative P3 amplitudes were evoked by the RIT condition than by the control condition.

#### LPC Component

**FIGURE 6:**
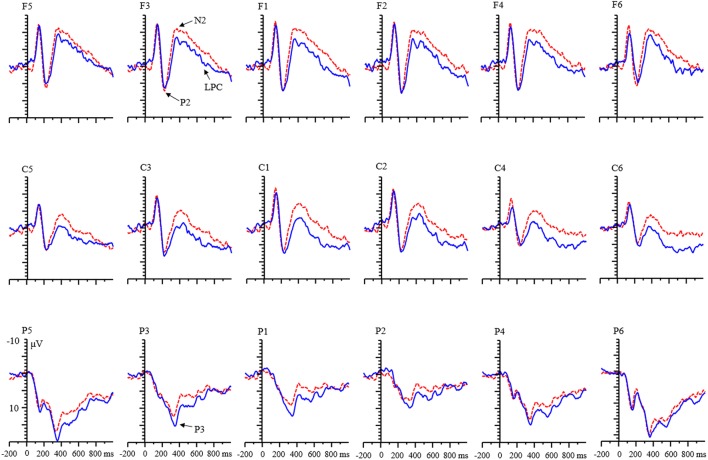
Grand averages of event-related potentials (ERPs) evoked by negative feedback for children.

With respect to the LPC component, a significant main effect of condition was found [*F*(1,68) = 55.15, *p* < 0.001, η^2^ = 0.45]. LPC amplitude for the RIT was more negative than that of control condition (**Figure 6**). There were no main effects of age and valence.

## Discussion

The current study examined the temporal course and neural correlates of the informative value of feedback in children and adults through a RIT. The behavioral results indicated that compared to adults, children in the RIT were less accurately to process the informative value of positive feedback and showed less flexibility to switch rules after receiving negative feedback. These results replicated previous finding that children are less successful in adjusting their behavior than adults, according to different feedbacks (Crone et al., 2004b; Huizinga et al., 2006; van Duijvenvoorde et al., 2008).

Consistent with related studies (Buchsbaum et al., 1974; Brecelj et al., 2002; Mai et al., 2011; Ferdinand et al., 2016), our ERP results showed that adults showed smaller N1 and P2 amplitudes than children did. The amplitude differences of these components might be associated with developmental of the efficiency of early visual processing, which was determined by brain maturation in the visual cortex system, including an increase in myelination (Steen et al., 1997) and synaptogenesis (Huttenlocher et al., 1982). Accordingly, children’s lower sensitivity to the informative value of feedback is possibly owing to the immaturity of the brain, particularly within the prefrontal cortices (Diamond, 2002; Sowell et al., 2004; Durston et al., 2006; van Duijvenvoorde et al., 2008). In addition, children’s internal control and monitoring processes are not yet fully developed. External feedback plays an important role in children’s behavioral control and they rely more on external feedback than adults (Crone et al., 2006b). Children usually need longer time to learn from feedback and are less skilled in using the information conveyed by the feedback to change their behavior (Hämmerer et al., 2011). Thus, the discrepancies in the sensitivity to informative value between the two age groups may also be due to difference in cognitive skills such as learning abilities (Smith et al., 2013).

The ERP results showed that, for either the positive or negative feedback, there was no significant differences in N1 amplitude of the two conditions. It was suggested that, regardless of the various types of conditions and feedbacks, the early attention on feedback was the same for children and adults (Zanolie et al., 2008a; Li et al., 2011; Huang et al., 2013).

The P2 amplitude in the RIT was more negative than that in the control condition. However, there was no such effect for negative feedback in the child group. The P2 component has been suggested to be related to visual processing during early stage, including attention assignation (Chen et al., 2007; San Martín et al., 2010; Huang et al., 2013) and feature detection (Thorpe et al., 1996; Bigman and Pratt, 2004; Nobre et al., 2006; Li et al., 2011). In the control condition, since feedback contains only valence, participants just needed to distinguish whether the choice was right or wrong. In contrast, participants in RIT condition had to pay attention to both the valence and the informative value of a given feedback. They needed to learn not only the correctness of their response but also the informative value of the feedback which would guide their subsequent rule searching. Therefore, participants assigned more attention resources to feedbacks in the RIT than they did in the control condition, which was reflected on the negative trend during the P2 time window in the RIT.

Interestingly, when children received a negative feedback, the difference between RIT and control condition during the P2 time window disappeared. There might be two possible reasons for this. First, children might dislike negative feedback or stimuli (Qu and Zelazo, 2007; van der Veen et al., 2008; Mai et al., 2011), which led them to pay less attention to the informative value of such kind of feedback or stimuli. Second, children’s abilities of inhibitory control and cognitive flexibility were still in development. When children confronted with negative feedback, they could not efficiently inhibit the wrong response and shift attention to the informative value of negative feedback (Crone et al., 2004a; Jansen et al., 2014). Studies about inhibitory control and task switch also indicated that compared to adults, children showed worse performance of inhibition and weaker switch ability (Satterfield et al., 1994; Davidson et al., 2006; Crone et al., 2008; Shing et al., 2010; Vara et al., 2014).

In line with previous findings, electrophysiological results showed that there was an obvious valence effect on the N2 component, except for the control condition in the child group (Hajcak et al., 2006; Holroyd et al., 2006). It was suggested that both children and adults can effectively distinguish positive and negative outcomes. With respect to the RIT task, negative feedback elicited a larger N2 than positive feedback did, possibly also reflecting the difference in processing different informative values of different types of feedback. That is, compared with the informative value of positive feedback, when participants received negative feedback, they needed to inhibit the invalid rule and shift to the possibly correct rules. Hence, participants had to pay much more attention to the negative feedback, which reflected on the larger N2. Additionally, relative to adults, children’s monitor system was still immature, so in the control condition, children could not yet differentiate between positive and negative feedback (Crone et al., 2006b; Eppinger et al., 2009; Mai et al., 2011).

During the N2 time window, both children and adults showed a larger N2 amplitude in the RIT than they did in the control condition. The N2 component is related to the monitoring of action, which in turn derives from the function of the anterior cingulate cortex (Van Veen and Carter, 2002; Botvinick et al., 2004; Chen et al., 2007, 2008; Gajewski et al., 2010; Finke et al., 2012; Leleu et al., 2012). Finke et al. (2012) suggested that the observed enhancement of target-locked N2 amplitudes for informative, cued trials might mirrored top-down control process. Similarly, a larger N2 amplitude in the RIT might reflect an increased top-down control when participants processing the informative value of feedback. That is, compared to the control condition, more efforts and resources were allocated to encode the informative value of feedback in the RIT compared to the control condition. Moreover, the increased N2 amplitude in the RIT might also reflect task preparation or anticipation (Folstein et al., 2008; Gajewski et al., 2010; Hsieh and Wu, 2011), because participants needed to prepare for the following rule selection after correctly extracting the information of rules from the first feedback in the RIT.

After the N2 component, a clear P3 component was observed for the adult group in both RIT and control condition; however, this component only appeared at the posterior sites for children. Squires et al. (1975) distinguished the P3 wave into two subcomponents, frontal P3a (300–400 ms) and posterior P3b (350–600 ms). Frontal P3a seems to be related to the shift of attention and may reflect the top-down process that switching attention to the incoming stimuli (Stige et al., 2007). Posterior P3b has been suggested to reflect task-relevant processes and context-updating (Barceló and Knight, 2002; Klingberg et al., 2002; Barceló, 2003, 2006; Crone et al., 2006b; Polich, 2007; Scisco et al., 2008; Periáñez and Barceló, 2009). In the present study, a posterior P3b was observed for both children and adults, implying that context updating was similar between these two age groups. The absence of frontal P3a for children might be due to the immature development of the prefrontal cortex, which was associated with the top-down control of attention on feedback (Klingberg et al., 2002; Crone et al., 2006c). Moreover, the importance of frontal P3a in processing the informative value of feedback is also consistent with the finding of Ferdinand et al. (2016), which demonstrated that adolescents with higher behavioral adjusting scores showed a more significant frontal P3a effect of feedback expectancy.

There was also a valence effect on the P3 component, as positive feedback elicited a larger P3 than negative feedback. However, this effect only existed in the adult group. The smaller P3 amplitudes to negative feedback may be associated with updating of working memory (Donchin and Coles, 1988; Watson et al., 2006). The positive feedback guides participants to maintain the correct rule in working memory. Compared to positive feedback, when participants received negative feedback, they needed to switch to a new rule, which leading a smaller P3. In contrast to adults, we did not find the valence effect in the child group. This might means that in children aged 8–10 years, the ability to update their working memory representation is still developing (Polich, 2007; Eppinger et al., 2009; Hämmerer et al., 2011).

Finally, the present study found that brain activation associated with the informative value of feedback was localized primarily at the left sites for children, and was not lateralized for adults. This result is consistent with previous findings (Doucet et al., 2005; Li et al., 2011). The developmental change from left lateralization to bi-lateralization in our study may reflect the development of cognitive flexibility, and top-down control on the processing of the informative value of feedback in rule learning. That is, in children, only left laterality of the brain was recruited in rule induction; in contrast, adults can use a wider brain network. Moreover, this difference may also reflect that children and adults adopt different strategies or skills to deal with the informative value of feedback (Sloutsky, 2010; Wolfensteller and von Cramon, 2011; Huang et al., 2013; Peters et al., 2014a). This is in line with the study of Huang et al. (2013). They designed an induction and non-induction tasks to investigate the neural correlates of age-related changes in category induction. The ERP results showed that the differences between the non-induction and the induction condition occurred predominantly in the left region for children, but not for adults. They inferred that children and adults may use different strategies in category induction. Recently, studies using neuroimaging method also found a significant difference in strategies used during feedback learning between children and adults, which were distinguishable at the neural level (Schmittmann et al., 2012; Peters et al., 2014b). Future studies may need to address whether children and adults in the RIT use different strategies in making use of the informative value to search for the correct rules.

## Conclusion

We used a RIT investigated the different brain potentials associated with the informative value of feedback between children and adults. The results showed that children were less effective at processing the informative value of feedback, particularly when they confronted with negative feedback. ERP results indicated that adults attended to the informative value of negative feedback at the P2 time window, whereas children did not show this effect until the later N2 time window. The informative value of feedback seems to be associated with the activation of the left-brain laterality for children, while bi-lateralized for adults, reflecting the maturation of brain.

## Ethics Statement

This study was carried out in accordance with the recommendations of Research Ethics Committee of Jiangxi Normal University. The protocol was approved by the Research Ethics Committee of Jiangxi Normal University.

## Author Contributions

FL designed the experiment and revised the paper. BD wrote the manuscript, collected and analyzed the data. BC contributed to revise the paper critically for suggestive comments on the initial version of this manuscript. WH contributed to the interpretation of the data for the work. All authors approved the final version of the manuscript and agreed to be accountable for all aspects of the work in ensuring that questions related to the accuracy or integrity of any part of the work are appropriately investigated and resolved.

## Conflict of Interest Statement

The authors declare that the research was conducted in the absence of any commercial or financial relationships that could be construed as a potential conflict of interest.
