# Supplementary material for: Different Electrophysiological Responses to Informative Value of Feedback Between Children and Adults
Source: Front Psychol. 2018 Apr 3;9:346. doi: 10.3389/fpsyg.2018.00346 (PMC5891721; doi:10.3389/fpsyg.2018.00346)
Supplement: Supplementary file 1 [file Table_1.PDF]

Supplementary

In the RIT, two of the four test stimuli shared two common attributes such as the shape and color with the target stimulus; while the other two shared only one common attribute, such as shape. For the purpose of the present study, we only analyzed the behavioral performance when children first selected the two-attributes shared stimuli. There were several correct logical routes and wrong routes in the RIT, and the accuracy here was defined as the correct logical routes, which was listed in the below table.

Table S1. The definition of accuracy of the performance of each trial in RIT.

<table>
<tr><th colspan="2">First selection</th><th colspan="2">Second selection</th><th colspan="2">Third selection</th><th rowspan="2">Accuracy</th></tr>
<tr><th>Stimulus selected</th><th>Feedback</th><th>Stimulus selected</th><th>Feedback</th><th>Stimulus selected</th><th>Feedback</th></tr>
<tr><td rowspan="7">Two-attributes shared stimuli</td><td rowspan="4">√</td><td rowspan="3">①</td><td>√</td><td colspan="2"></td><td>Right</td></tr>
<tr><td rowspan="2">×</td><td rowspan="2"></td><td>√</td><td>Right</td></tr>
<tr><td>×</td><td>Error</td></tr>
<tr><td>②</td><td>×</td><td colspan="2"></td><td>Error</td></tr>
<tr><td rowspan="3">×</td><td rowspan="3"></td><td rowspan="2">√</td><td rowspan="2"></td><td>√</td><td>Right</td></tr>
<tr><td>×</td><td>Error</td></tr>
<tr><td>×</td><td colspan="2"></td><td>Error</td></tr>
<tr><td>One-attributes shared stimuli</td><td colspan="6">Not listed here</td></tr>
</table>

Note: ① indicate that the second selection shares one or two common attributes with first selection; ② indicate that the second selection shares none common attribute with first selection;
